# Acetyl-CoA synthetase 2 induces pyroptosis and inflammation of renal epithelial tubular cells in sepsis-induced acute kidney injury by upregulating the KLF5/NF-κB pathway

**DOI:** 10.1186/s12964-024-01556-3

**Published:** 2024-03-21

**Authors:** Jian Lu, Ya Hou, Si-Xiu Liu, Bo Jin, Jing Liu, Nan Li, Yan Zhu, Qing-Yan Zhang, Cheng Wan, Yuan Feng, Jun Xie, Chun-Ming Jiang

**Affiliations:** 1 Department of Nephrology, the Affiliated Hospital of Medical School, Nanjing Drum Tower Hospital, Nanjing University, Jiangsu Province, Nanjing, 210008 China; 2 Department of Cardiology, the Affiliated Hospital of Medical School, Nanjing Drum Tower Hospital, Nanjing University, Jiangsu Province, Nanjing, 210008 China; 3 Medical School, Nanjing University, Jiangsu Province, Nanjing, 210093 China

**Keywords:** Acetyl-CoA synthetase 2, Pyroptosis, Sepsis, Acute kidney injury

## Abstract

**Background:**

Pyroptosis of the renal tubular epithelial cells (RTECs) and interstitial inflammation are central pathological characteristics of acute kidney injury (AKI). Pyroptosis acts as a pro-inflammatory form of programmed cell death and is mainly dependent on activation of the NLRP3 inflammasome. Previous studies revealed that acetyl-CoA synthetase 2 (ACSS2) promotes inflammation during metabolic stress suggesting that ACSS2 might regulate pyroptosis and inflammatory responses of RTECs in AKI.

**Methods and results:**

The expression of ACSS2 was found to be significantly increased in the renal epithelial cells of mice with lipopolysaccharide (LPS)-induced AKI. Pharmacological and genetic strategies demonstrated that ACSS2 regulated NLRP3-mediated caspase-1 activation and pyroptosis through the stimulation of the KLF5/NF-κB pathway in RTECs. The deletion of ACSS2 attenuated renal tubular pathological injury and inflammatory cell infiltration in an LPS-induced mouse model, and ACSS2-deficient mice displayed impaired NLRP3 activation-mediated pyroptosis and decreased IL-1β production in response to the LPS challenge. In HK-2 cells, ACSS2 deficiency suppressed NLRP3-mediated caspase-1 activation and pyroptosis through the downregulation of the KLF5/NF-κB pathway. The KLF5 inhibitor ML264 suppressed NF-κB activity and NLRP3-mediated caspase-1 activation, thus protecting HK-2 cells from LPS-induced pyroptosis.

**Conclusion:**

Our results suggested that ACSS2 regulates activation of the NLRP3 inflammasome and pyroptosis by inducing the KLF5/NF-κB pathway in RTECs. These results identified ACSS2 as a potential therapeutic target in AKI.

**Graphical Abstract:**

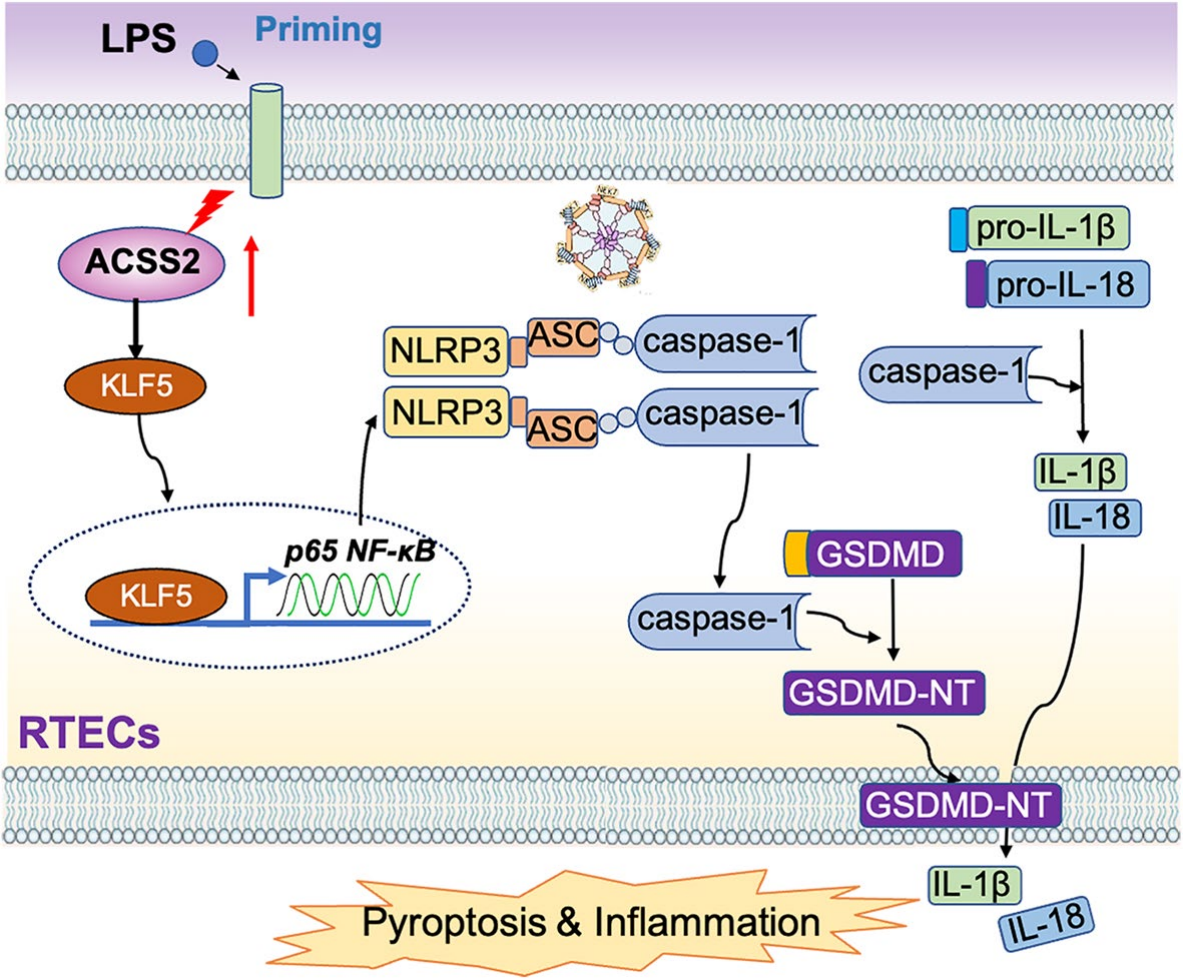

We found that the expression of ACSS2 was significantly increased in RTECs in septic AKI. ACSS2-deficient mice displayed resistance to renal damage in this model. ACSS2 regulated NLRP3-mediated caspase-1 activation and pyroptosis through the KLF5/NF-κB pathway in RTECs. Pharmacological inhibition of KLF5 suppressed NLRP3 activation and caspase-1 cleavage by downregulating of NF-κB. Our results suggest that ACSS2 is a pro-pathogenic mediator of cell pyroptosis of RTECs and renal inflammation in sepsis-induced AKI.

## Introduction

Acute kidney injury (AKI) is characterized by an acute decline of renal function and is associated with high morbidity and mortality [1]. Incomplete recovery from AKI is associated with poor prognosis and can even involve the development of end-stage renal disease. Although AKI is a well-known complication of sepsis [2, 3], the underlying pathophysiological mechanisms remain poorly understood.

Renal tubular epithelial cells (RTECs) play a key role in the development of AKI, as injury to and death of these cells, along with renal interstitial inflammation, are key pathological characteristics of AKI [4]. Notably, the death of RTECs is known to occur as a result of AKI, but the death of these cells is also a vital pathogenic factor in the development of AKI, as irreversible injury and cell death of RTECs directly leads to the obstruction of renal function recovery and the poor prognosis associated with AKI.

Pyroptosis, a form of programmed cell death, has been mechanistically linked with renal inflammation in AKI [5, 6]. Pyroptosis is characterized by the swelling and rupture of the plasma membrane, the release of inflammatory factors, and inflammatory cell infiltration; accordingly, this form of cell death contributes to inflammatory responses. The process of pyroptosis is triggered by the activation of the canonical NLRP3 inflammasome and non-canonical inflammasomes, and it leads to the upregulation of caspase-1/11 (caspase-4 in humans) [7].

Gasdermin D (GSDMD) is a specific substrate of the inflammatory caspase cascade [8, 9]. Caspase activation leads to the hydrolysis of GSDMD at specific sites to produce amino-terminal and carboxyl-terminal fragments [10]. The GSDMD N-terminal fragment (GSDMD-N) migrates to the plasma membrane and forms pores that ultimately promote cell death [10, 11]. However, the mechanism of pyroptosis in promoting the development of renal injury in sepsis-induced AKI has not been fully demonstrated.

Recently, acetyl-CoA synthetase 2 (ACSS2) has attracted extensive research interest in multiple contexts. ACSS2 synthesizes acetyl-CoA to support cytosol lipid synthesis and contributes to metabolic reprogramming via histone epigenetic modulation [12]. ACSS2 has been shown to be essential for the epigenetic regulation of lipogenic gene expression [13] under hypoxic stress to support tumor survival and growth [14, 15], and ACSS2-mediated metabolic reprogramming provides additional nutrients for macropinocytosis in pancreatic cancer [16]. In addition to synthesizing acetyl-CoA from acetate, ACSS2 can also convert butyrate into butyryl-CoA, which has been found to promote the activity of carnitine palmitoyltransferase 1A and fatty acid oxidation, thereby promoting inducible regulatory T cell differentiation and immune homeostasis [17]. These observations position ACSS2 as a critical link between metabolism and inflammation, which provoked us to explore whether it might also be an important contributor to the pathogenesis of sepsis. We, therefore, sought to study the effect of ACSS2 on inflammatory responses in sepsis-induced AKI.

In the current study, we aimed to clarify the role of ACSS2 in the pathogenesis of AKI. We showed that the expression of ACSS2 was significantly increased in RTECs from septic mice. ACSS2-deficient mice were resistant to the renal damage and inflammation associated with LPS-induced sepsis. In terms of the mechanism by which ACSS2 contributed to the induction of RTEC injury by sepsis, we demonstrated that ACSS2 regulates NLRP3-mediated caspase-1 activation and pyroptosis through the KLF5/NF-κB pathway. ACSS2-deficient mice displayed reduced NLRP3 inflammasome activation, reduced production of IL-1β, and reduced pyroptosis of RTECs, and similar results were observed using an in vitro model. Importantly, the pharmacologic inhibition of KLF5 suppressed NLRP3 activation and cleavage of caspase-1 by downregulating NF-κB. Overall, our results suggested that ACSS2 is a mediator of pyroptotic cell death of RTECs and renal inflammation in sepsis-induced AKI.

## Material and methods

### Reagents and antibodies

Lipopolysaccharides (*Escherichia coli* O111:B4, L2630, for animal experiments; *Escherichia coli* O111:B4, L4391, for cell experiments) were purchased from Merck (New Jersey, USA). ACSS2 inhibitor (cat no: S8588) was purchased from Selleck (Houston, USA). ML264 (cat no: HY-19994) was purchased from MedChemExpress (New Jersey, USA). Lotus Tetragonolobus Lectin (LTL) (cat no: FL-1321-2) was purchased from Thermo Fisher (MA, USA).

The following antibodies were used. ACSS2 antibody (cat no: ab133664) from Abcam (Cambridge, UK); KIM-1 antibody (cat no: sc-518008) from Santa Cruz (California, USA); F4/80 antibody (cat no: 28463-1-AP) from Proteintech (Wuhan, China); Cleaved GSDMD (N Terminal) antibody (cat no: A22523) and NLRP3 antibody (cat no: A5652) from Abclonal (Wuhan, China); caspase-1 antibody (cat no: 24232), anti-IL-1β antibody (cat no: 12242) from Cell Signaling Technology (Massachusetts, USA); GSDMD antibody (cat no: AF4012), NF-κB p65 antibody (cat no: AF5006), phospho-NF-κB p65 (Ser536) antibody (cat no: AF2006), and KLF5 antibody (cat no: AF7542) from Affinity (Changzhou, China).

### Animal experiments

All animal experimental protocols were approved by the Ethics Committee on Laboratory Animal Management of the Nanjing Drum Tower Hospital, the Affiliated Hospital of Nanjing University Medical School (Approval number: 2022AE01023). All animals received humane care following the National Research Council's guidelines for the Care and Use of Laboratory Animals. All animal experiments complied with ARRIVE guidelines.

C57BL/6J-background mice homozygote for genetic deletion of the *Acss2* gene (Strain NO: T017245) were obtained from GemPharmatech Co., Ltd (Nanjing, China). Male C57BL/6J littermates served as controls. For the establishment of the sepsis-induced AKI mice model [18], LPS (10 mg/kg) or vehicle was injected intraperitoneally into the mice after adaptive feeding for 1 week. The mice were sacrificed 24 hours after injection, and blood and kidney samples of mice were collected.

### Blood urea nitrogen (BUN) and serum creatinine measurements

Blood samples of mice were incubated at room temperature for 30 minutes, and serum was obtained by centrifugal separation at 3000 rpm for 10 minutes. The levels of BUN (Jiancheng, C013-2-1, Nanjing, China) and serum creatinine (Jiancheng, C011-2-1, Nanjing, China) were determined using a kit according to the manufacturer’s instructions.

### Histological analyses

Kidney tissues were fixed in 4% formaldehyde, embedded in paraffin, and then sectioned. Hematoxylin-eosin (H&E) staining was performed to identify pathological changes in the kidney.

### Cell culture

HK-2 cells were purchased from ATCC. Cells were cultured in DMEM/F12 medium with 10% fetal bovine serum and penicillin (100 IU/mL)/streptomycin (100 μg/mL). According to preliminary experiments, ACSS2 inhibitor (ACSS2i) was used at concentrations of 10 μmol/L or 20 μmol/L, and ML264 was used at a concentration of 10 μmol/L. Cells were challenged with LPS or an equivalent amount of vehicle (DMSO) for 24 or 48 hours.

### Transfection of ACSS2 siRNA

HK-2 cells at 70 to 80% confluence were transfected with ACSS2 siRNA using lipofectamine 2000 (cat no: 11668019, ThermoFisher, MA, USA) according to the manufacturer’s instructions. The ACSS2 siRNA and a corresponding scrambled siRNA was purchased from GenePharma (Shanghai, China). The sequence of the siRNA targeting human ACSS2 was 5’-CCT TCC ACA AAT ACG GAA ATT-3'.

### RT-qPCR

Total RNA was isolated using TRIzol reagent and then reverse-transcribed to synthesize cDNA using a commercial cDNA synthesis kit (Vazyme, R323-01, Nanjing, China). The generated cDNA was amplified by using appropriate primers and SYBR Green PCR Master Mix assay kit (Vazyme, Q711-02, Nanjing, China). PCR reactions were performed using an ABI PRISM 7300 System.

### Western blotting

Total protein was extracted from HK-2 cells and kidney tissues with RIPA buffer. Protein concentrations were determined using the BCA assay, and equal amounts of lysate proteins were separated with SDS-PAGE and transferred to PVDF membranes. Membranes were blocked with 5% bovine serum albumin and then incubated overnight at 4 ℃ with primary antibodies diluted according to the manufacturers’ instructions. After being washed with TBS buffer containing 0.1% Tween, the membranes were incubated with a horseradish peroxidase-conjugated secondary antibody. Membranes were incubated with ECL reagent, and signal was visualized using a chemiluminescence digital imager.

### Immunofluorescence staining

Paraformaldehyde-fixed HK-2 cells were permeabilized with 0.1% Triton X-100. Formalin-fixed, paraffin-embedded kidney sections and HK-2 cells were then blocked with 5% BSA and incubated sequentially with primary and secondary antibodies. All immunofluorescence images were obtained with a confocal microscope (Olympus, FV3000).

### Transmission electron microscopy (TEM)

HK-2 cells were fixed with 2.5% glutaraldehyde for 5 minutes at room temperature. The cells were then gently scraped in one direction with a cell scraper to avoid scratching. The cell suspension was centrifuged at 3000 rpm for 2 minutes, and the pelleted cells were fixed in 2.5% glutaraldehyde at 4 ℃ overnight, post-fixed in tetroxide osmium, sequentially dehydrated in ethanol, and embedded in a mixture of acetone and resin. Kidney tissue was sliced into ultrathin sections and stained with uranyl acetate and lead citrate. Samples were subsequently imaged by TEM as previously described [19].

### Statistical analysis

Results were expressed as the mean ± standard deviation (SD). Statistical significance among more than two experimental groups was assessed using one-way ANOVA. Statistical analysis was performed with Prism version 7.0 (GraphPad Software Inc., San Diego, CA, USA). A value of *P* < 0.05 was considered statistically significant.

## Results

### The NLRP3 inflammasome and pyroptosis are activated in an LPS-induced mouse sepsis model

**Fig. 1:**
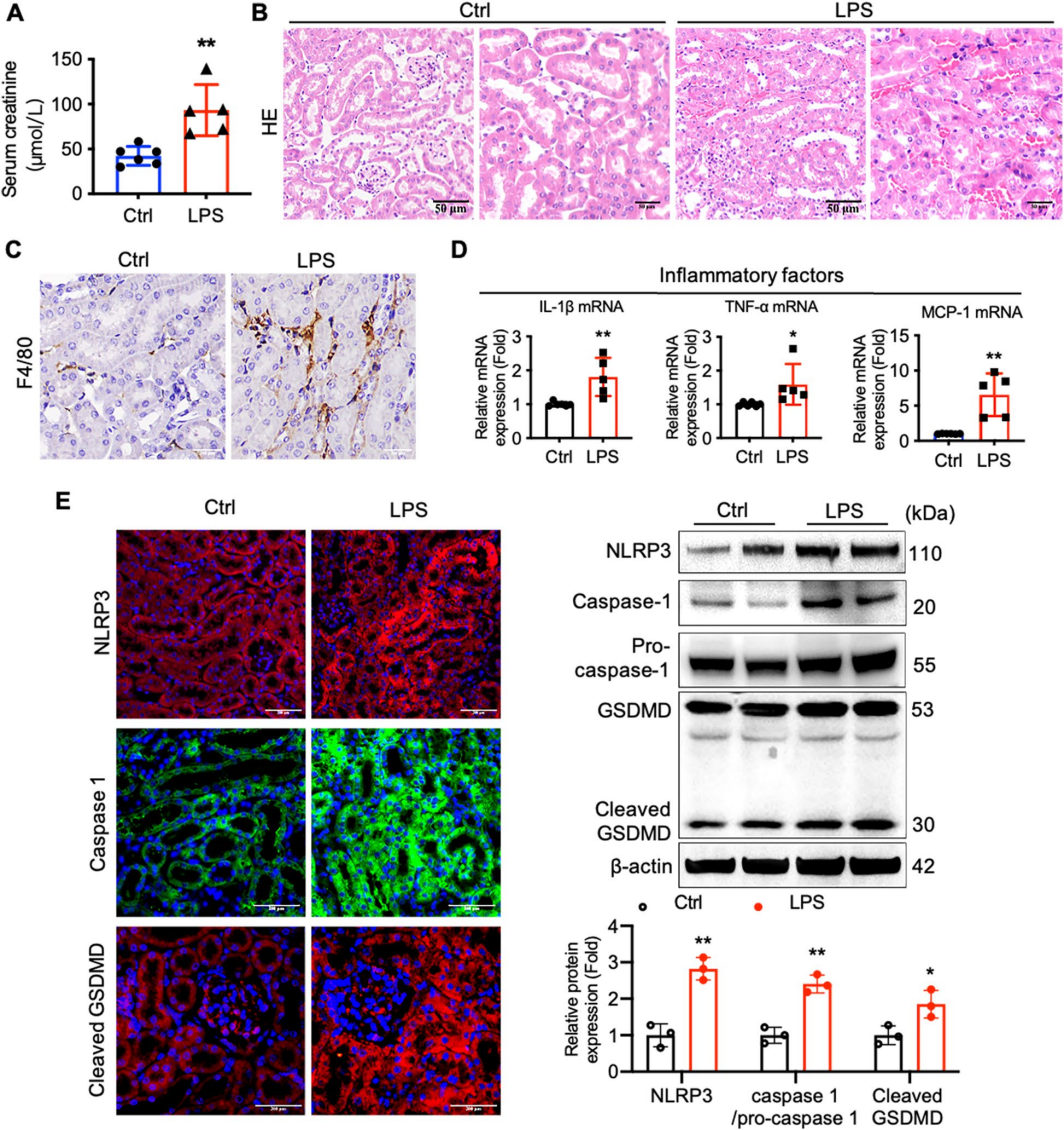
The NLRP3 inflammasome and pyroptosis are activated in an LPS-induced mouse sepsis model. **A** Serum creatinine levels in vehicle-treated mice and LPS-induced sepsis mice were measured (*n* = 5-6). **B** H&E staining of kidney tissues from the AKI model mice and control mice (Scale bars, 50 μm). **C** F4/80 staining of kidney tissues of the noted mice (Scale bars, 200 μm). **D **Expression of pro-inflammatory factors at the mRNA level in the kidneys of vehicle- or LPS-treated mice were determined using RT-qPCR (*n* = 5). **E** The protein expression of NLRP3, cleaved GSDMD, and caspase-1 in the kidney were detected by immunofluorescent staining (green, caspase-1; red, NLRP3 or cleaved GSDMD; blue, DAPI) and Western blotting (*n* = 3). Data were presented as mean ± SD. ^*^P < 0.05, ^**^P < 0.01 *vs.* Ctrl

An in vivo model of AKI was established by treating mice with LPS injection to induce sepsis. This treatment led to decreased renal function as shown by higher serum creatinine levels in mice treated with LPS as compared to mice treated with vehicle (Fig. 1A). Morphologically, mice challenged with LPS displayed an impaired renal tubular structure as compared with mice in the vehicle-treated group; in particular, the LPS-treated mice exhibited swelling and cytoplasmic vacuolation of RTECs and brush border loss (Fig. 1B). Staining for the macrophage-specific marker F4/80 indicated higher infiltration of macrophages in the kidneys of LPS-treated mice (Fig. 1C). The levels of transcription of genes encoding the pro-inflammatory cytokines IL-1β, TNF-α, and MCP-1 were found to be higher in the kidneys of LPS-treated mice (Fig. 1D). Taken together, these results indicated that severe inflammatory reactions were induced in the kidneys of LPS-challenged mice.

In addition, in response to the LPS challenge, renal tissues exhibited morphological manifestations that are typical of pyroptosis, including the swelling and rupture of the cell membrane, cell vacuolation, and the secretion of multiple pro-inflammatory cytokines. In order to further investigate pyroptotic activity in the mouse model, we quantified the levels of proteins known to be involved in pyroptosis, including cleaved caspase-1 and GSDMD in kidneys of model and control mice. In addition, since the activation of the NLRP3 inflammasome is considered essential in the process leading to pyroptosis, we explored the expression of NLRP3 as well. According to both Western blotting and immunofluorescence assays, the levels of these three pyroptosis-related proteins in the kidney tissues of LPS-challenged mice were significantly increased relative to control (Fig. 1E), providing further evidence consistent with a model in which pyroptosis was involved in the pathogenesis of renal epithelial tubular injury in the LPS-induced sepsis mouse model.

### ACSS2 contributes to inflammation in LPS-treated HK-2 cells

**Fig. 2:**
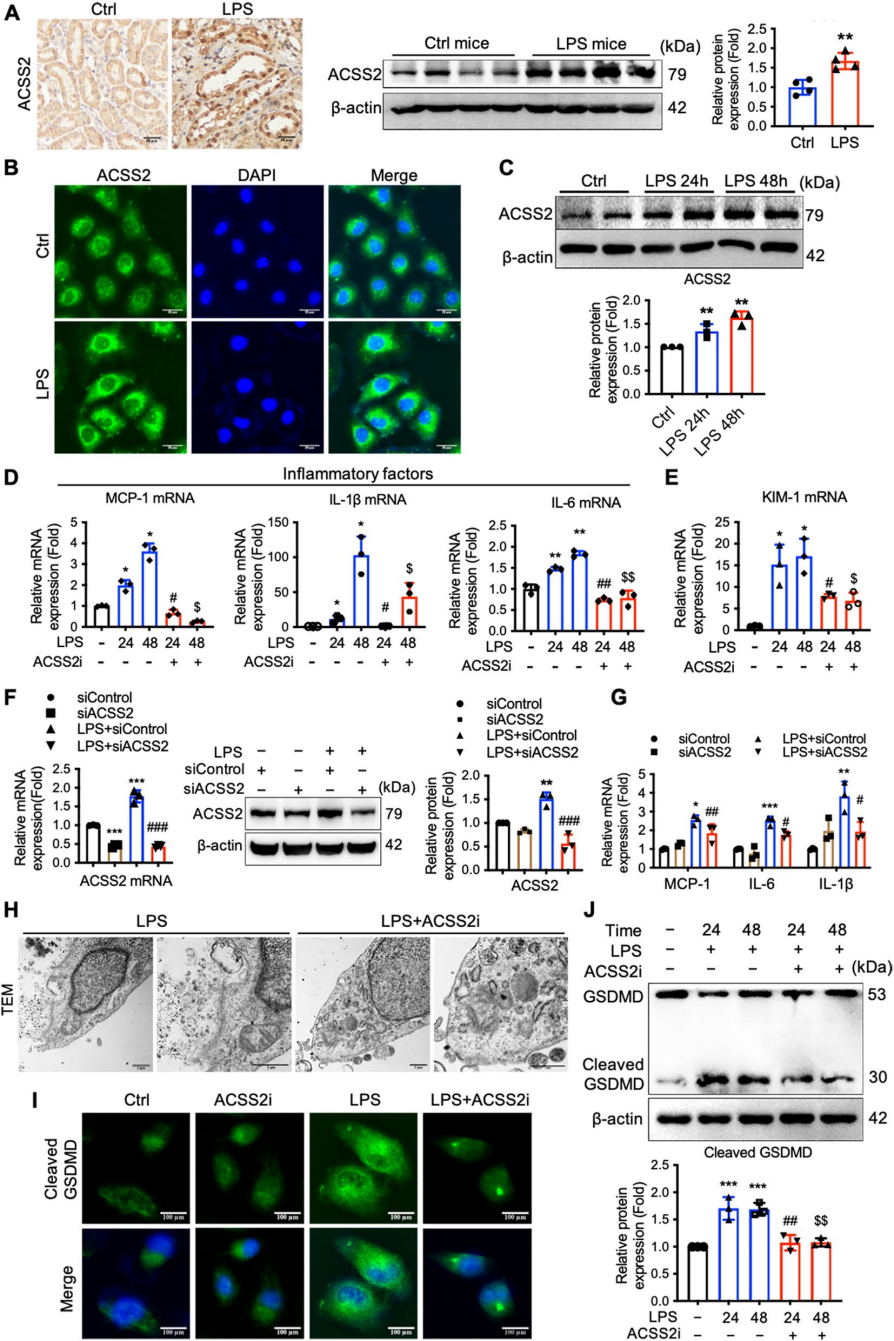
ACSS2 contributes to the inflammatory response and pyroptotic cell death in LPS-treated HK-2 cells. **A** Representative immunohistochemical staining and Western blotting analysis of ACSS2 in the kidneys of control and LPS-induced AKI mice. (Scale bars, 50 μm; *n* = 4). **B** Immunofluorescent staining of ACSS2 in HK-2 cells with or without LPS treatment (green, ACSS2; blue, DAPI; scale bars, 200 μm). **C** Western blotting analysis of ACSS2 protein in HK-2 cells treated with LPS for 24 or 48 hours (*n* = 3). **D** Relative mRNA expression levels of the pro-inflammatory cytokines (MCP-1, IL-6, and IL-1β) in HK-2 cells with or without pre-treatment with an ACSS2 inhibitor (ACSS2i) prior to LPS treatments for 24 or 48 hours (*n* = 3). **E** Relative mRNA expression level of kidney injury molecule-1 (KIM-1) in HK-2 cells with or without pre-treatment with ACSS2i prior to LPS treatments for 24 or 48 hours (*n* = 3). **F** The mRNA and protein levels of ACSS2 in HK-2 cells transfected with an ACSS2-specific siRNA or scrambled siRNA prior to LPS treatments for 24 hours (*n* = 3). **G** Relative mRNA expression levels of the pro-inflammatory cytokines (MCP-1, IL-6, and IL-1β) in HK-2 cells transfected with an ACSS2-specific siRNA or scrambled siRNA prior to LPS treatments for 24 hours (*n* = 3). H: TEM observation of the ultrastructure of HK-2 cells with or without pre-treatment with ACSS2i prior to LPS treatments for 24 hours (Scale bars, 5 μm). I: Immunofluorescence detection of the expression of cleaved GSDMD in HK-2 cells with or without LPS (1 μg/mL) and with or without co-treatment with ACSS2i (10 μmol/L) for 24 hours (Scale bars, 100 μm). J: Western blotting analysis of GSDMD in HK-2 cells which were stimulated with LPS (1 μg/mL) or vehicle and co-incubated with ACSS2i (10 μmol/L) for 24 or 48 hours (*n* = 3). Data were presented as mean ± SD. ^*^P < 0.05, ^**^P < 0.01, ^***^P < 0.005 *vs.* Ctrl; ^#^*P* < 0.05, ^##^*P* < 0.01, ^###^*P* < 0.005 *vs.* LPS or LPS + siControl; ^$^*P* < 0.05 *vs.* LPS for 48 hours

We previously demonstrated that ACSS2 is overexpressed following renal tubular injury and that it promotes cellular deterioration in diabetic renal tubular injury. In the present study, we also found that the level of ACSS2 protein was increased in RTECs of LPS-induced sepsis mice relative to RTECs from vehicle-treated mice (Fig. 2A). Similarly, the expression of ACSS2 protein was increased upon LPS stimulation of cultured HK-2 cells (Fig. 2B, C).

To further clarify whether ACSS2 contributed to the inflammatory responses to sepsis in kidney cells, we investigated the influence of ACSS2 activity on the ability of LPS to increase expression of pro-inflammatory cytokines in HK-2 cells. For these experiments, we pre-treated cells with an ACSS2 inhibitor (ACSS2i; 10 or 20 μmol/L) or transfected an ACSS2-specific siRNA prior to treatment with LPS. Here, it was found that the mRNA levels of the pro-inflammatory cytokines MCP-1, IL-1β, and IL-6, and the renal injury biomarker KIM-1 were increased in HK-2 cells upon LPS treatment, and pretreatment with ACSS2i partially rescued these changes (Fig. 2D, E). LPS stimulation was also found to increase expression of ACSS2, but transfection with an ACSS2-specific siRNA significantly attenuated this stimulatory effect (Fig. 2F). The knockdown of expression of ACSS2 decreased expression of the three inflammatory cytokines (Fig. 2G). This protection of RTECs from LPS-mediated inflammation by pharmacological and genetic inhibition of ACSS2 is consistent with a model in which ACSS2 overexpression is a factor leading to renal injury in sepsis.

To investigate the interaction of ACSS2 with pyroptosis in this process, we first used TEM to probe ultra-morphological changes to cultured cells. We found that HK-2 cells treated with LPS displayed morphological characteristics typical of pyroptosis, including cell swelling and vacuolation and membrane pore formation; these changes were ameliorated by pre-treatment of the cells with ACSS2i (Fig. 2H).

We also used immunofluorescence and Western blotting to probe the cleavage status of GSDMD, which functions as a key effector downstream of caspase-1 activation. Active caspases generated an N-terminal cleavage product of GSDMD, called cleaved GSDMD, which mediates cell membrane pore formation to drive pyroptosis. We observed that, in addition to increasing pyroptosis, LPS stimulation also increased cleavage of GSDMD, and we found that pharmacological inhibition of ACSS2 attenuated the effects of LPS on GSDMD cleavage (Fig. 2I, J). Taken together, these results suggested that ACSS2 enhances NLRP3 inflammasome activity and the cleavage of GSDMD that is associated with pyroptosis in RTECs.

### Promotion of NLRP3 inflammasome activation and GSDMD cleavage by ACSS2 depends on NF-κB signaling activation

**Fig. 3:**
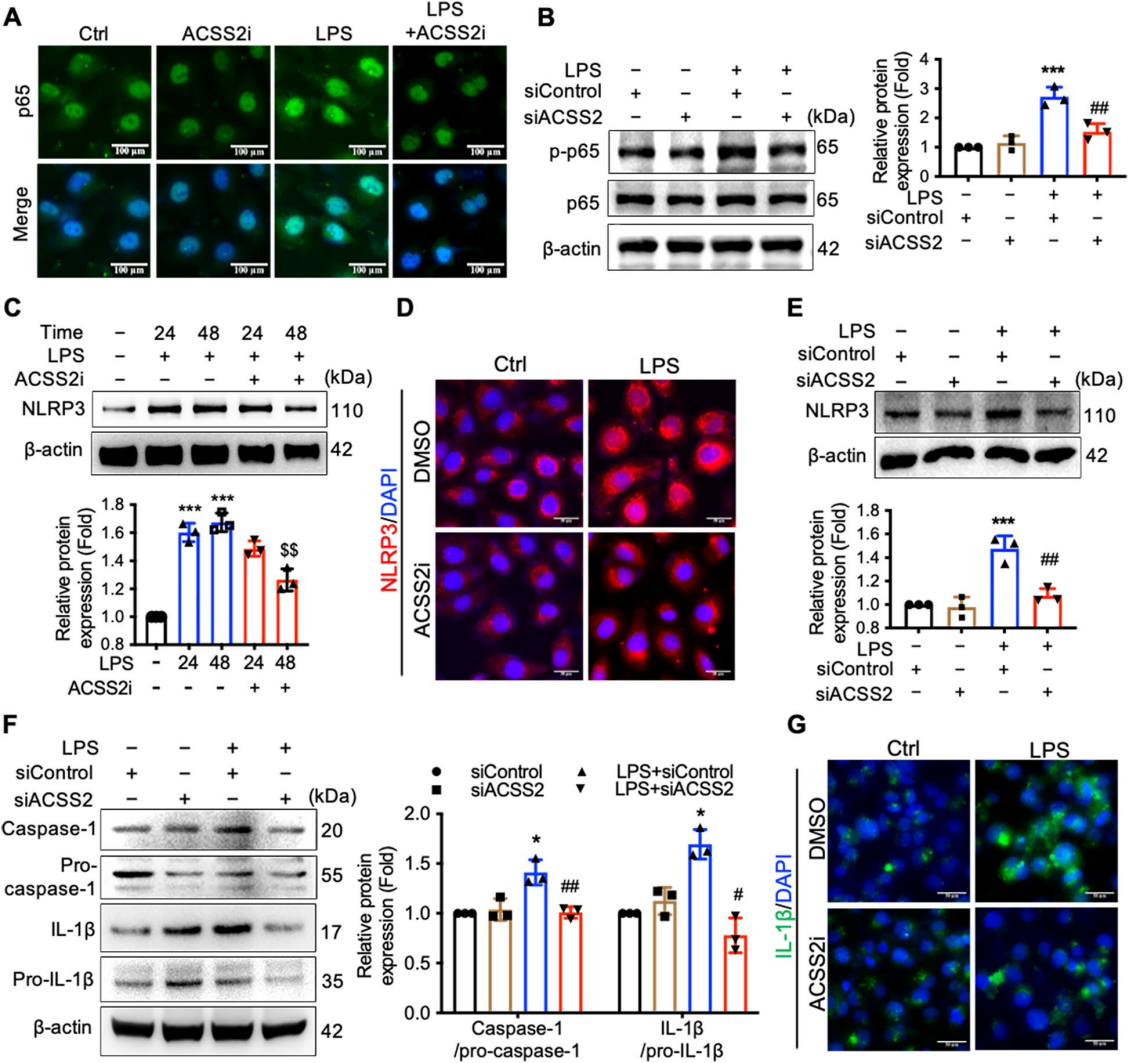
Promotion of NLRP3 inflammasome activation and GSDMD cleavage by ACSS2 depends on NF-κB signaling activation. **A** The immunofluorescence staining of NF-κB p65 was detected in HK-2 cells which were treated with LPS (1 μg/mL) or vehicle along with ACSS2i (10 μmol/L) for 24 hours. (green, p65; blue, DAPI; scale bars, 100 μm). **B** The effects of ACSS2 siRNA (siACSS2) on the protein expression and phosphorylation of p65 in HK-2 cells stimulated with LPS (1 μg/mL) were analyzed by Western blotting (*n* = 3). **C** and **D** Western blotting and immunofluorescence analyses of NLRP3 in HK-2 cells which were stimulated with LPS (1 μg/mL) or vehicle and co-incubated with ACSS2i (10 μmol/L) for 24 or 48 hours (red, NLRP3; blue, DAPI, scale bars, 100 μm; *n* = 3). **E** and **F** Western blotting analyses of the protein expression of (**E**) NLRP3 or (**F**) pro-caspase-1, caspase-1, pro-IL-1β, and IL-1β in HK-2 cells which were treated with LPS (1 μg/mL) or vehicle and ACSS2 siRNA (siACSS2) for 24 hours (*n* = 3). **G** The expression of IL-1β in mouse kidney was determined by immunofluorescence (green, IL-1β; blue, DAPI; scale bars, 50 μm). Data were presented as mean ± SD. ^*^P < 0.05, ^***^P < 0.005 *vs.* control; ^#^*P* < 0.05, ^##^*P* < 0.01 *vs.* LPS or LPS + siControl

Two steps have been identified in the stimulation of the NLRP3 inflammasome. The first step involves the transcriptional and translational production of inflammasome components, especially via stimulation of NLRP3 expression by the transcription factor NF-κB. The second step involves the driving of NLRP3 oligomerization upon the identification of indications of cell damage, such as K^+^ efflux or the presence of extracellular ATP [20]. Thus, we next aimed to determine the effects of ACSS2 knockdown on NF-κB signaling activation and the expression of NLRP3 in HK-2 cells treated with LPS. Upon stimulation of HK-2 cells with LPS, we observed an increase in the phosphorylation and nuclear translocation of the p65 subunit of NF-κB (Fig. 3A, B). We also observed a corresponding increase in NLRP3 expression upon LPS stimulation, and this increase was mitigated by knockdown of ACSS2 expression (Fig. 3C-E), suggesting that decreasing ACSS2 activity inhibited activation of the NLRP3 inflammasome. We also found that the levels of mature caspase-1 and IL-1β were markedly higher under LPS stimulation, and these LPS-stimulated levels were reduced by decreasing ACSS2 activity pharmacologically or with a genetic knockdown (Fig. 3F, G). These results suggested that ACSS2 promotes the activation of the NLRP3 inflammasome by activating NF-κB.

### ACSS2 ablation protects kidney function and ameliorates renal tubular injury in mice with LPS-induced acute kidney injury

**Fig. 4:**
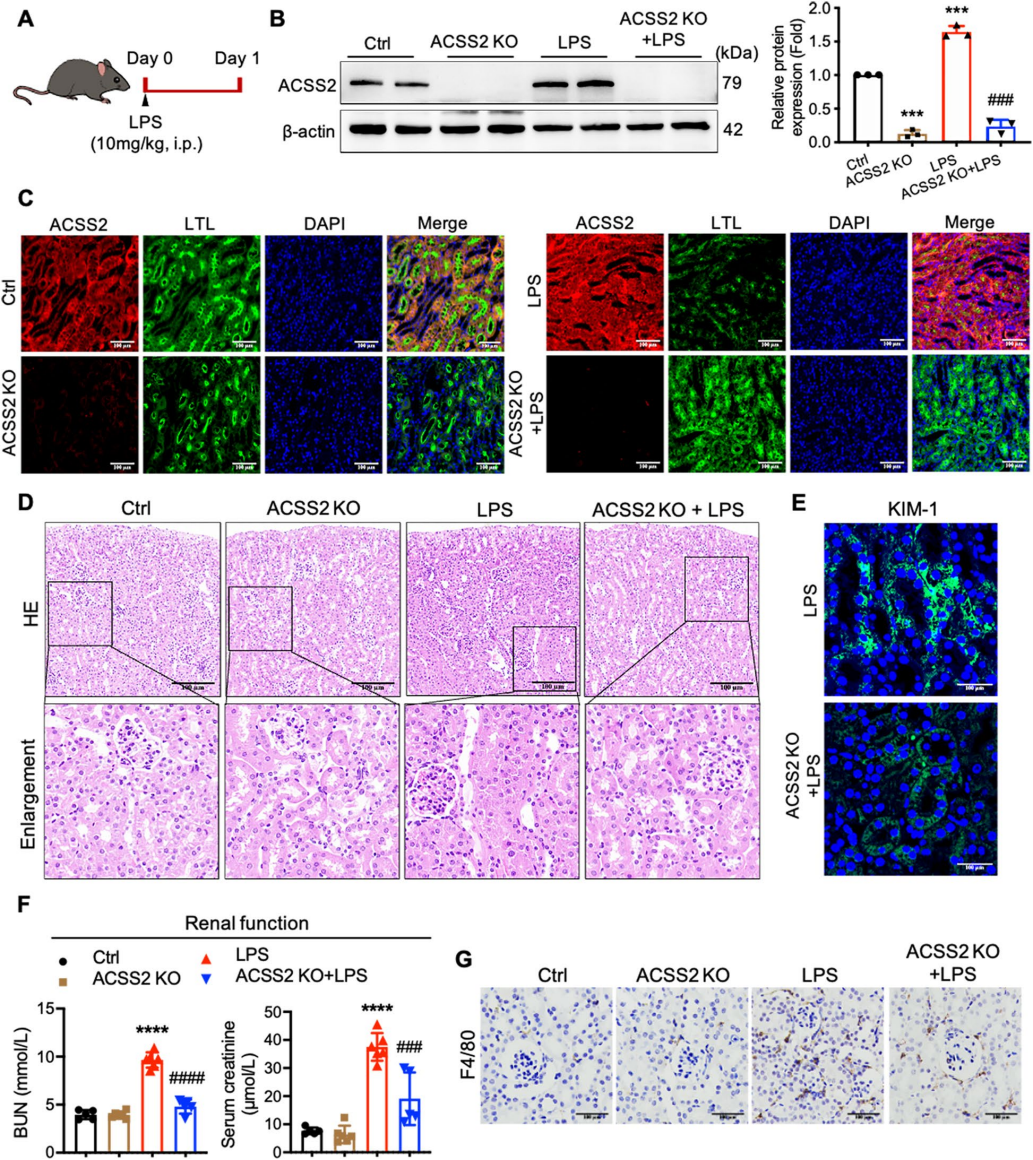
ACSS2 ablation protects kidney function and ameliorates renal tubular injury in LPS-induced mice. **A** Schematic diagram of the strategy leading to the LPS-induced sepsis mouse model. **B** Western blotting analyses of the protein expression levels of ACSS2 in renal cortex of mice (*n* = 3). **C** Representative immunofluorescent images of ACSS2 and Lotus Tetragonolobus Lectin (LTL) in the kidney tissues from mice (Scale bars, 200 μm). **D** and **E** Representative images of **(D)** H&E staining and (**E**) immunofluorescent staining of KIM-1 in the kidneys of mice (Scale bars, 100 μm). **F** The serum levels of BUN and serum creatinine were measured (*n* = 5-6). **G** The expressions of F4/80 (a marker for macrophages) in the kidneys were analyzed (Scale bars, 100 μm). Data were presented as mean ± SD. ^*^P < 0.05, ^****^P < 0.0001 *vs.* Ctrl; ^#^*P* < 0.05, ^##^*P* < 0.01, ^###^*P* < 0.005 *vs.* LPS

Because our in vitro studies established a detrimental effect of ACSS2 activity on cell injury and inflammation response in RTECs, we next investigated the pathological contribution of ACSS2 in the in vivo model of septic renal tubular injury. We challenged both ACSS2 KO and control mice with an injection of LPS (10 mg/kg, i.p.) and compared their responses to those of littermates injected with vehicle (Fig. 4A). Blood samples and kidney tissues were collected 24 hours after LPS exposure.

The effects of LPS challenge on ACSS2 expression, renal function, histopathology of kidney tissues, and the inflammation response in renal tubular tissues were determined. According to Western blotting analyses, LPS treatment was found to increase the protein level of ACSS2 in the kidneys of wild-type mice, but it failed to increase ACSS2 expression in the kidneys of ACSS2^−/−^ mice (Fig. 4B). Similarly, when the distribution of ACSS2 protein in RTECs from vehicle-treated wild-type mice was investigated using immunofluorescence, it was found to be primarily localized to the cytoplasm, but its expression was dramatically increased upon LPS challenge. Furthermore, the results of Western blotting and immunofluorescence analyses confirmed the deletion of the ACSS2 gene in ACSS2^−/−^ mice (Fig. 4C).

As shown by H&E staining, wild-type mice treated with LPS exhibited renal tubular injury characterized by renal tubular cell swelling, loss of brush border, and vacuolization (Fig. 4D), and the renal tubular injury biomarker kidney injury molecule-1 (KIM-1) was expressed at a higher level in these mice (Fig. 4E). However, the LPS-induced renal tubular injury (Fig. 4D) and KIM-1 expression (Fig. 4E) were both dramatically attenuated in ACSS2^−/−^ mice. This protection against renal damage was reflected in the protection against LPS-induced renal dysfunction by genetic deletion of the ACSS2 gene, as reflected by decreased serum levels of BUN and serum creatinine in ACSS2^−/−^ mice relative to wild-type mice challenged with LPS (Fig. 4F). In addition, LPS-stimulated interstitial inflammation, as demonstrated by increased inflammatory cell infiltration, was observed in LPS-treated wild-type mice, but this process was also mitigated in ACSS2^−/−^ mice (Fig. 4G). These data indicated that the deletion of ACSS2 mitigated renal tubular injury and the interstitial inflammation response.

### ACSS2 deletion decreases GSDMD-mediated cell pyroptosis in RTECs of LPS-induced mice

**Fig. 5:**
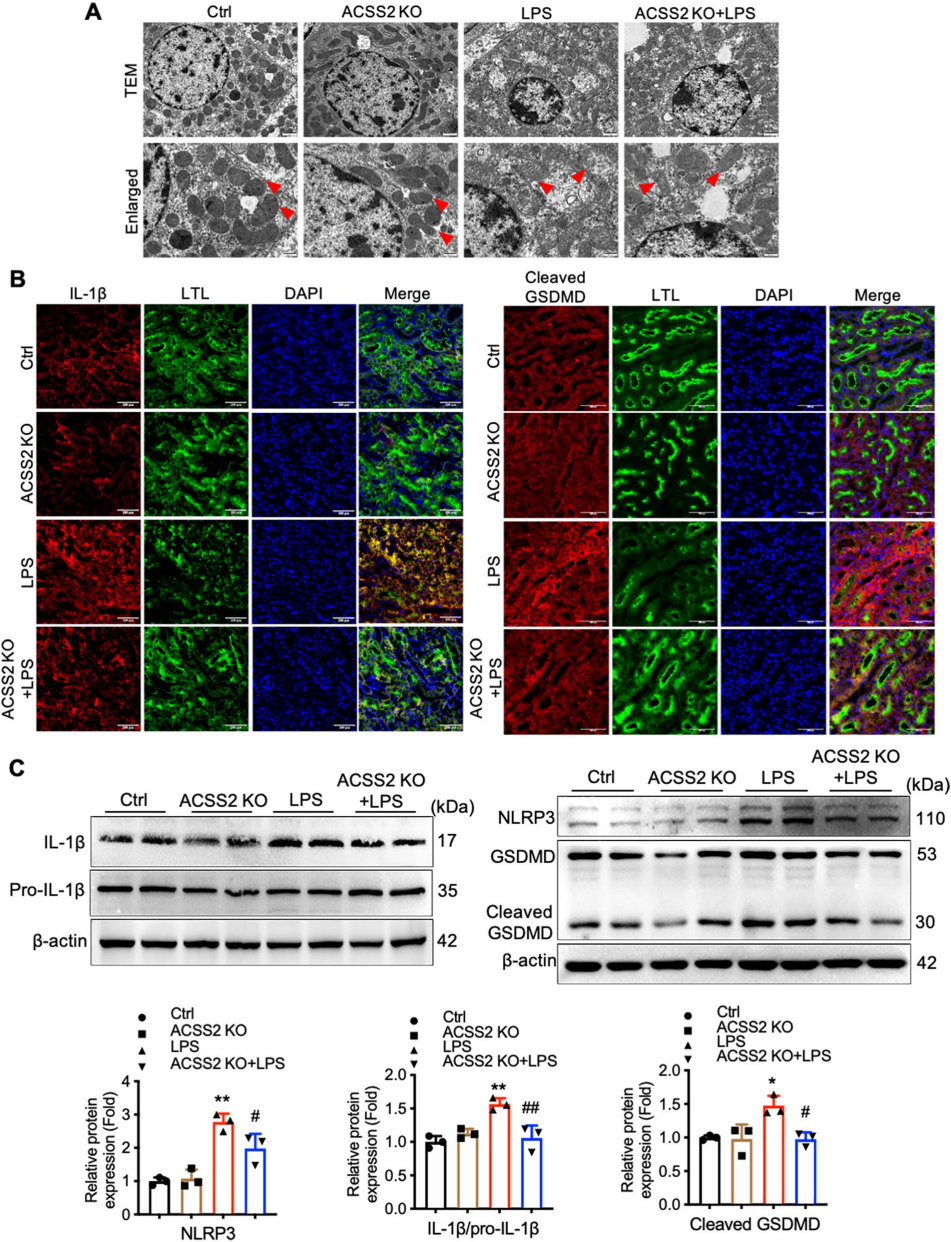
ACSS2 deletion decreases inflammasome activation and GSDMD-mediated cell pyroptosis in renal tubular cells of LPS-induced mice. **A** Representative transmission electron microscopy (TEM) images of kidney tissues (Scale bars, 1 μm or 0.5 μm). **B** Representative immunofluorescent staining of cleaved GSDMD and IL-1β in the kidneys (green, LTL; red, IL-1β or cleaved GSDMD; blue, DAPI; Scale bars, 200 μm). **C **Western blotting analyses of the protein expression levels of NLRP3, IL-1β, and the cleavage of GSDMD in the kidney tissues of the noted mice (*n* = 3). Data were presented as mean ± SD. ^*^P < 0.05, ^***^P < 0.005 *vs.* Ctrl; ^#^*P* < 0.05, ^##^*P* < 0.01 *vs.* LPS

In order to investigate the mechanisms of cellular damage in the kidney tissue of LPS-treated mice, we performed morphological analyses of kidney tissues from mice treated with LPS or vehicle. As shown in Fig. 5A, the RTECs of wild-type mice treated with LPS were characterized by cell swelling, vacuolation, and membrane pore formation, all evidence of pyroptosis, while the RTECs isolated from ACSS2^−/−^ mice treated with LPS did not exhibit these morphological features.

In order to more specifically analyze pyroptotic activity on the molecular level, Western blotting and immunofluorescence assays were used to quantify the expression of proteins associated with pyroptosis as well as the cleavage status of caspase-1. In RTECs from wild-type mice, LPS treatment was found to correlate with increased cleavage of caspase-1 as well as increased expression of NLRP3, IL-1β, and GSDMD. However, in ACSS2-/- mice, the LPS-induced elevations of the protein levels of NLRP3, caspase-1, and IL-1β were lower (Fig. 5B, C). Therefore, we concluded that ACSS2 deletion mitigated the activation of the NLRP3 inflammasome and decreased pyroptosis in RTECs of the mouse LPS-induced sepsis model.

### ACSS2 induces KLF5-mediated p65 activation and downstream cell pyroptosis in LPS-induced kidney tubular injury

**Fig. 6:**
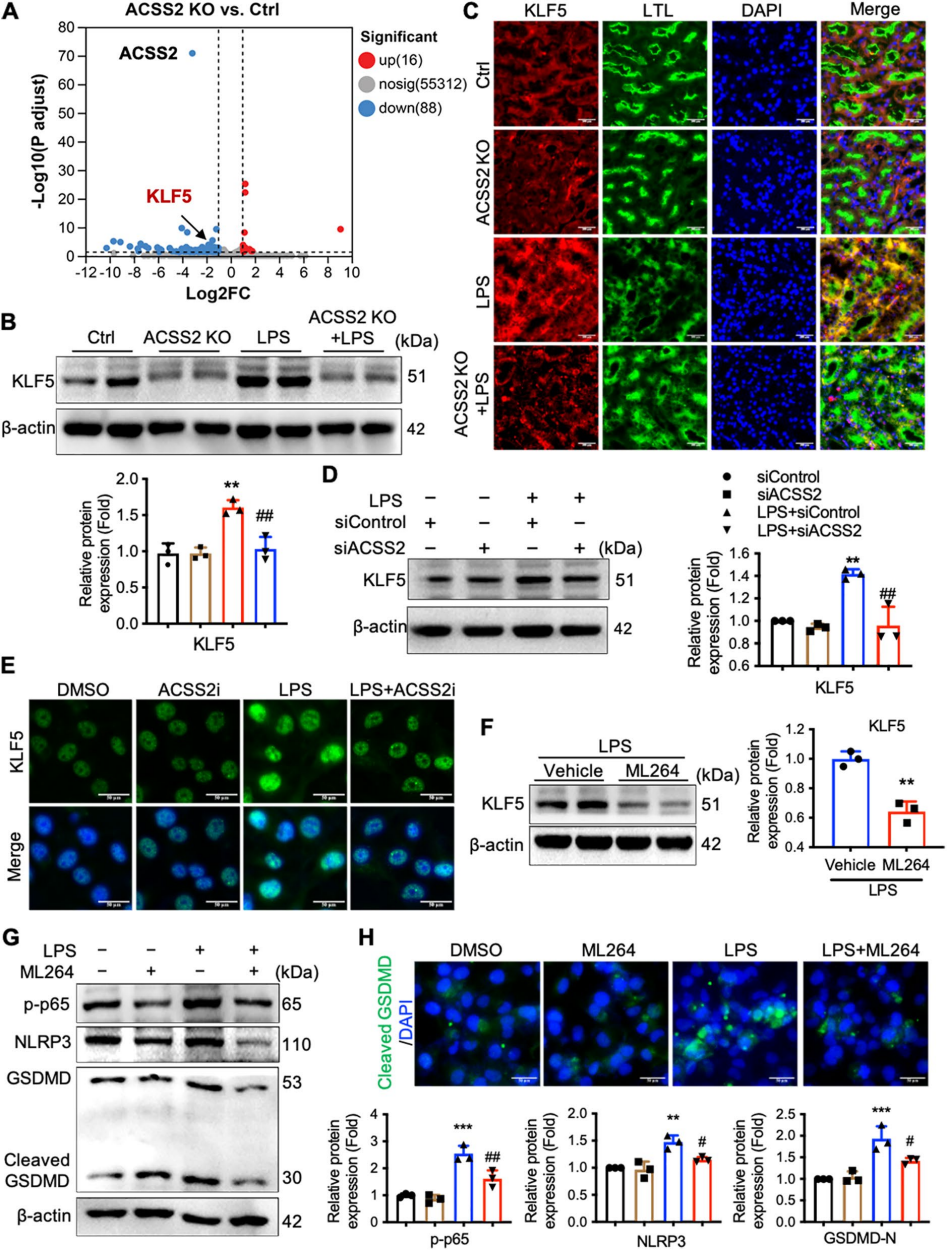
ACSS2 induces KLF5-mediated p65 activation and downstream cell pyroptosis in LPS-induced kidney tubular injury. **A** RNA-seq analysis of kidney tissues from ACSS2 gene knockout (KO) mice and wild-type mice was performed. The colored dots indicated differentially expressed genes (DEGs). **B** Relative protein expression levels of KLF5 in the mouse kidney (*n* = 3). **C** Representative immunofluorescent images of KLF5 and LTL in the renal cortex of mice (green, LTL; red, KLF5; blue, DAPI; Scale bars, 100 μm). **D** Western blotting was used to compare expression of KLF5 in HK-2 cells treated with LPS (1 μg/mL) or vehicle and with ACSS2 siRNA or scrambled siRNA for 24 hours (*n* = 3). **E** Immunofluorescence staining was used to detect KLF5 (green, KLF5; blue, DAPI; scale bars, 50 μm). **F** and **G** HK-2 cells were pre-treated with ML264 (10 μmol/L) or vehicle for 1 hour, then with LPS (1 μg/ml) or vehicle for 24 hours. Western blotting was used to analyze **(F)** the expression of KLF5 or** (G)** the phosphorylation of p65, the expression of NLRP3, and the cleavage of GSDMD (*n* = 3). **H** The levels of cleaved GSDMD were determined by immunofluorescence staining (green, cleaved GSDMD; blue, DAPI; scale bars, 50 μm). Data were presented as mean ± SD. ^*^P < 0.05, ^***^P < 0.005 *vs.* Ctrl, siControl or LPS + vehicle; ^#^*P* < 0.05, ^##^*P* < 0.01 *vs.* LPS + siControl or LPS + vehicle

To further probe the mechanisms by which ACSS2 modulates pyroptosis in the establishment of kidney damage in the mouse LPS-induced sepsis model, we performed RNA sequencing (RNA-seq) and bioinformatics analyses. In our RNA-seq analyses, we identified multiple differentially expressed genes (DEGs) in the kidney between wild-type mice and ACSS2-/- mice (Fig. 6A). We were particularly interested in the differential expression of the gene encoding the transcription factor Krüppel Like Factor 5 (KLF5), which was recently identified to be overexpressed in RTECs of the septic AKI model [21]. In addition, this transcription factor has been reported to mediate the activity of activated NF-κB [22, 23].

We further explored the possibility that ACSS2 regulates NLRP3 activation and pyroptosis by influencing the expression of KLF5. Western blotting analyses showed that LPS treatment of wild-type mice induced an increased expression of KLF5 in kidney tissues; however, ACSS2 deficiency attenuated the LPS-induced increased expression of KLF5 (Fig. 6B, C).

In the cultured-cell system, treatment of HK-2 with LPS led to an increase in KLF5 protein expression, which was suppressed by pharmacological inhibition or gene knockdown of ACSS2 (Fig. 6D, E). Therefore, we investigated the potential relationship between KLF5 activity and LPS-stimulated pyroptosis and inflammation in HK-2 cells through the use of ML264, a selective pharmacological inhibitor of KLF5 activation (Fig. 6F). Pre-treatment of ML264 in HK-2 cells prior to LPS stimulation was found to alleviate the LPS-mediated transactivation of NF-κB p65, as manifest in phosphorylation of Ser536, and this pre-treatment consequently reduced the activation of NLRP3 and pyroptosis (Fig. 6G, H). Thus, we concluded that ACSS2 promotes pyroptotic cell death of RTECs in the sepsis-induced model of AKI via the KLF5/NF-κB pathway.

## Discussion

The mechanisms of pathogenesis leading to the death of RTECs and tubular interstitial inflammation in AKI are incompletely understood. Pyroptosis is known to be important in the death of RTECs, which is an indispensable characteristic of AKI. The major finding in the present study was that ACSS2 was found to act as a critical regulator of activation of the NLRP3 inflammasome and pyroptosis in RTECs in vitro and in vivo LPS-induced sepsis models of AKI. We further displayed a novel molecular mechanism whereby the transcription factor KLF5 served as a downstream target molecule for the pro-inflammatory action of ACSS2. In addition, we found that ACSS2-dependent and KLF5-mediated NF-κB activation is critical for the activation of the NLRP3 inflammasome. Thus, we provided evidence supporting the pathophysiological relevance of ACSS2 in mediating inflammatory responses of RTECs in AKI.

ACSS2 is highly expressed in multiple mammalian cells such as cortical neuron cells, brown adipocytes, hepatic cells, and renal cells [24]. ACSS2 has been implicated in numerous pathophysiological conditions, including inflammation, metabolic disorders, and cancer. For example, the activation of the mTORC2 signaling pathway has been reported to promote the formation of acetyl-CoA by ACSS2, thereby driving brown adipogenesis [25]. In addition, ACSS2 was shown to mediate the reprogramming of the synthesis and utilization of fatty acids in a way that supports the tumorigenic properties of cancer cells. ACSS2 can facilitate de novo acetate production from pyruvate, expanding its role in alternative carbon metabolic pathways for maintaining acetyl-CoA pools [26]. However, to this point, few studies have investigated the pathophysiological relationship between ACSS2 and pyroptosis in sepsis-induced kidney injury.

We found that ACSS2 expression was shown to be positively correlated with the expression of key component molecules of the NLRP3 inflammasome in the renal tubules of LPS-treated mice, which suggested that ACSS2 might be involved in the pathogenesis of AKI. While mice in which ACSS2 had been genetically deleted had an apparently normal renal phenotype under control conditions, these mice experienced an amelioration of renal dysfunction in the context of sepsis-induced AKI, accompanied by a mitigation of both renal tubular injury and renal interstitial inflammation. Notably, hypoxia and glucose deprivation have been shown to stimulate ACSS2 signaling in the tumor microenvironment [27, 28]. Considering that hypoxia is a hallmark of septic-AKI, this connection may explain the upregulation of ACSS2 in RTECs during AKI.

Emerging evidence has implicated pyroptosis in the pathogenesis of AKI and in the activation of pro-inflammatory cascades and renal injury [29, 30], although the regulation of pyroptosis remains unclear. The gasdermin family has been shown to contain key executors of the cell pyroptosis process, and a proteolytic fragmentation of GSDMD has been found to be a main mediator of pyroptosis of RTECs during sepsis-induced AKI [31]. In this study, we discovered that the regulatory role for ACSS2 in pyroptosis and inflammation in RTECs during AKI likely involves modulation of GSDMD cleavage. For example, mice genetically lacking ACSS2 exhibited decreased cleavage of GSDMD in RTECs during the induction of sepsis, suggesting that GSDMD is a downstream effector of ACSS2 in the process of cell pyroptotic death during sepsis-induced AKI.

KLF5 has been demonstrated to regulate tubulointerstitial inflammation in renal diseases [21]. KLF5 knockdown has been shown to inhibit the phosphorylation of NF-κB p65, consequently ameliorating LPS-induced AKI. Previous studies have also shown that KLF5 participates in LPS-mediated inflammation and cellular damage of the lung [32] and kidney [33], but a pathophysiological role of KLF5 in the process of pyroptosis had not yet been demonstrated. We found in this study that the levels of expression of KLF5 were increased in RTECs upon LPS treatment, and the pharmacological or genetic loss of ACSS2 activity suppressed KLF5 expression. Moreover, inhibition of KLF5 activation by ML264 suppressed the transactivation of NF-κB p65 via the inhibition of its phosphorylation at the Ser536 site and consequently reduced the activation of NLRP3 and cell pyroptotic death.

Taken together, the results of our study demonstrated that ACSS2 regulates NLRP3 activation and pyroptosis in the context of LPS-induced RTEC injury through the activation of the KLF5/NF-κB pathway. This finding suggests that further study into the deteriorating effects of ACSS2 in inflammatory diseases is warranted.

## Data Availability

All data associated with this study are presented in the paper. Raw data can be obtained from the corresponding authors upon reasonable request.
